# Imaging the Antistaphylococcal Activity of CATH-2: Mechanism of Attack and Regulation of Inflammatory Response

**DOI:** 10.1128/mSphere.00370-17

**Published:** 2017-11-01

**Authors:** Viktoria A. F. Schneider, Maarten Coorens, Johanna L. M. Tjeerdsma-van Bokhoven, George Posthuma, Albert van Dijk, Edwin J. A. Veldhuizen, Henk P. Haagsman

**Affiliations:** a Department of Infectious Diseases and Immunology, Division of Molecular Host Defence, Faculty of Veterinary Medicine, Utrecht University, Utrecht, The Netherlands; b Department of Cell Biology, Cell Microscopy Core, University Medical Center Utrecht, Utrecht, The Netherlands; University of Nebraska Medical Center

**Keywords:** *Staphylococcus aureus*, antimicrobial peptides, cathelicidins, electron microscopy, host defense peptides, inflammation, live imaging, macrophages

## Abstract

Chicken cathelicidin-2 (CATH-2) is a broad-spectrum antimicrobial host defense peptide (HDP) that may serve as a paradigm for the development of new antimicrobial agents. While previous studies have elucidated the mechanism by which CATH-2 kills *Escherichia coli*, its mode of action against Gram-positive bacteria remains to be determined. In this study, we explored the underlying antibacterial mechanism of CATH-2 against a methicillin-resistant strain of *Staphylococcus aureus* and the effect of CATH-2-mediated *S. aureus* killing on immune activation. Visualization of the antimicrobial activity of CATH-2 against *S. aureus* with live-imaging confocal microscopy demonstrated that CATH-2 directly binds the bacteria, which is followed by membrane permeabilization and cell shrinkage. Transmission electron microscopy (TEM) studies further showed that CATH-2 initiated pronounced morphological changes of the membrane (mesosome formation) and ribosomal structures (clustering) in a dose-dependent manner. Immunolabeling of these sections demonstrated that CATH-2 binds and passes the bacterial membrane at subminimal bactericidal concentrations (sub-MBCs). Furthermore, competition assays and isothermal titration calorimetry (ITC) analysis provided evidence that CATH-2 directly interacts with lipoteichoic acid and cardiolipin. Finally, stimulation of macrophages with *S. aureus* and CATH-2 showed that CATH-2 not only kills *S. aureus* but also has the potential to limit *S. aureus*-induced inflammation at or above the MBC. Taken together, it is concluded that at sub-MBCs, CATH-2 perturbs the bacterial membrane and subsequently enters the cell and binds intracellular *S. aureus* components, while at or above the MBC, CATH-2 causes disruption of membrane integrity and inhibits *S. aureus*-induced macrophage activation.

**IMPORTANCE** Due to the high use of antibiotics in both human and veterinary settings, many bacteria have become resistant to those antibiotics that we so heavily rely on. Methicillin-resistant *S. aureus* (MRSA) is one of these difficult-to-treat resistant pathogens for which novel antimicrobial therapies will be required in the near future. One novel approach could be the utilization of naturally occurring antimicrobial peptides, such as chicken CATH-2, which have been show to act against a wide variety of bacteria. However, before these peptides can be used clinically, more knowledge of their functions and mechanisms of action is required. In this study, we used live imaging and electron microscopy to visualize in detail how CATH-2 kills *S. aureus*, and we investigated how CATH-2 affects immune activation by *S. aureus*. Together, these results give a better understanding of how CATH-2 kills *S. aureus* and what the potential immunological consequences of this killing can be.

## Abstract (precis)

Due to the high use of antibiotics in both human and veterinary settings, many bacteria have become resistant to those antibiotics that we so heavily rely on. Methicillin-resistant *S. aureus* (MRSA) is one of these difficult-to-treat resistant pathogens for which novel antimicrobial therapies will be required in the near future. One novel approach could be the utilization of naturally occurring antimicrobial peptides, such as chicken CATH-2, which have been show to act against a wide variety of bacteria. However, before these peptides can be used clinically, more knowledge of their functions and mechanisms of action is required. In this study, we used live imaging and electron microscopy to visualize in detail how CATH-2 kills *S. aureus*, and we investigated how CATH-2 affects immune activation by *S. aureus*. Together, these results give a better understanding of how CATH-2 kills *S. aureus* and what the potential immunological consequences of this killing can be.

## INTRODUCTION

The extensive use of antibiotics over past years has led to an increase in the number of difficult-to-treat antibiotic-resistant pathogens (1–3). In the search for novel anti-infective therapies against these pathogens, host defense peptides (HDPs) have been suggested as potential candidates (4). HDPs are small effector molecules of the innate host defense system and have been shown to be crucial in the protection against various infections (5–7). Their described functions range from broad-spectrum direct antimicrobial activity (8) to immunomodulatory activities, such as neutralization of lipopolysaccharide (LPS) (9) and enhancement of DNA-induced Toll-like receptor (TLR) activation (10, 11).

Chicken cathelicidin-2 (CATH-2) is an HDP with strong antimicrobial potential (12). This 26-amino-acid peptide is highly cationic and amphipathic, with salt-insensitive antibacterial activity against various Gram-positive and Gram-negative bacteria (13, 14). In addition, CATH-2 has a proline-induced kink that was demonstrated to play an important role in the biological activity of the peptide (12, 15). Furthermore, CATH-2 exhibits several immunomodulatory properties, such as the neutralization of LPS and enhancement of DNA-induced macrophage activation (10, 16).

So far, various different antimicrobial mechanisms have been described for different HDPs, including membrane disruption or killing by inhibition of important cell functions (protein folding and nucleic acid and protein synthesis) (8, 17, 18). Recently, we visualized and characterized the bactericidal effects of CATH-2 on *Escherichia coli* and demonstrated that CATH-2 has distinctive effects on Gram-negative bacterial membranes and intracellular contents at concentrations that do not immediately kill the bacteria (19). In addition, the killing of *E. coli* by CATH-2 was shown to be immunologically silent, preventing the inflammatory response against neutralized bacteria (20). However, the mechanism of action of CATH-2 against Gram-positive bacteria has not been studied yet. This study focuses on the antibacterial activity of CATH-2 against methicillin-resistant *Staphylococcus aureus* (MRSA). By using different imaging techniques, such as live-imaging confocal microscopy and (immuno-)transmission electron microscopy [(immuno-)TEM], the antibacterial actions of CATH-2 were visualized and characterized. With these techniques, we observed that at subminimal bactericidal concentrations (sub-MBCs), CATH-2 already enters the bacteria and induces membrane ruffling and intracellular morphological changes, including ribosomal structural changes, while at or above the MBC, CATH-2 binds and permeabilizes the *S. aureus* membrane. Furthermore, lipoteichoic acid (LTA) and cardiolipin were found to directly interact with CATH-2 through ionic interaction, suggesting these could be the initial targets of the peptide on the bacterial surface. Finally, analysis of macrophage activation showed that CATH-2 not only kills *S. aureus* but also dampens *S. aureus*-induced macrophage activation at or above the MBC compared to its effects at sub-MBCs.

## RESULTS

### CATH-2 rapidly kills *S. aureus.*

**FIG 1:**
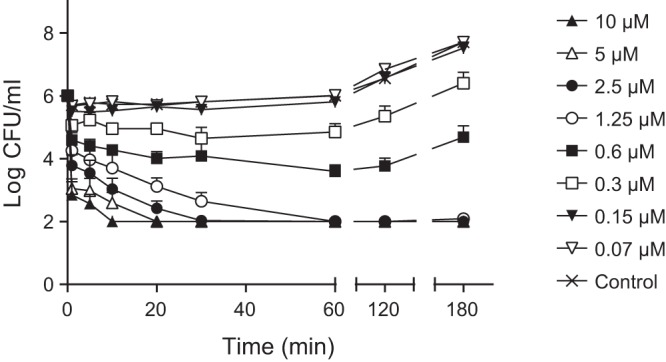
CATH-2 rapidly kills *S. aureus*. *S. aureus* was incubated at 37°C with CATH-2 at the indicated concentrations, and aliquots were serially diluted and spread plated at 1, 5, 10, 30, 60, 120, and 180 min. After 16 h at 37°C, viable bacteria were counted. Data represent three independent measurements (mean values ± standard errors of the means [SEM]).

The bactericidal activity of CATH-2 against *S. aureus* was assessed by colony-counting assays. The MBC of CATH-2 against *S. aureus* (at 2 × 10^6^ CFU/ml) was 5 µM (data not shown). Additional killing kinetics demonstrated that the peptide rapidly kills *S. aureus*, with 10 µM CATH-2 killing all bacteria within 10 min (Fig. 1). Due to the different experimental setup compared to a conventional colony-counting assay, the MBC was 1.25 µM, showing complete bacterial killing at 60 min. While lower peptide concentrations were able to hamper bacterial growth, bacteria recovered and started to regrow after 60 min.

### CATH-2 targets the septum and permeabilizes the bacterial membrane.

**FIG 2:**
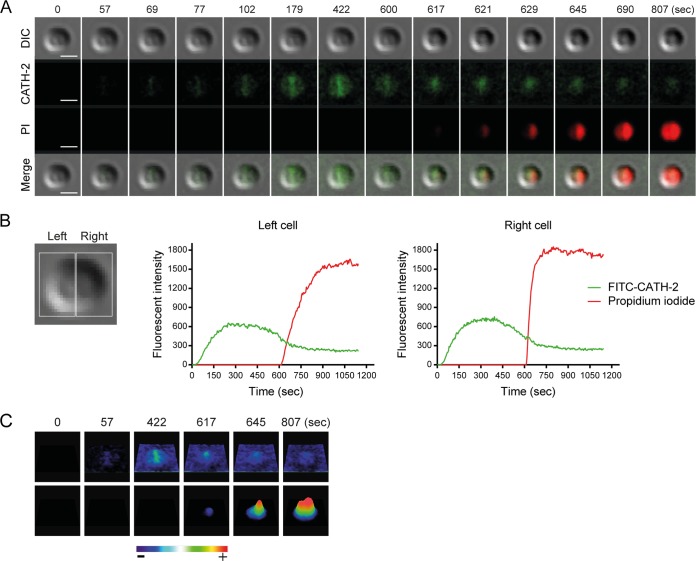
Time-lapse imaging demonstrates binding of CATH-2 to *S. aureus* and permeabilization of membranes. (A) Peptide binding (green), entry of PI (red), and cell shrinkage (differential inference contrast [DIC]) are shown at various time points. Bars = 1 µm. (B) Fluorescence intensities of FITC–CATH-2 (0.5 µM) and PI (2.5 µM) were measured in a dividing cell (shown as left and right cell). (C) Heat intensity plots demonstrate localization of FITC–CATH-2 and PI.

To visualize the interaction between CATH-2 and *S. aureus*, live imaging with fluorescein isothiocyanate (FITC)-labeled CATH-2 was performed. This showed that the peptide preferentially binds to the septum, as indicated by higher local fluorescence intensities (Fig. 2A; Movie S1 and S2 in the supplemental material). This was followed by permeabilization of the bacteria, as observed by propidium iodide (PI) staining of the bacterial DNA. Simultaneously with the permeabilization, bacterial shrinkage occurred. Compared to the size of the cells at the start of the experiment (0 s), peptide treatment caused 0.15-µm shrinkage of the cells at 807 s (Fig. 2A). Single-cell analysis was carried out to determine fluorescence intensity levels and showed first CATH-2 binding, followed after a few minutes by intracellular PI staining (Fig. 2B). In addition, heat intensity plots confirmed the preferential binding of the peptide at the bacterial septum (Fig. 2C).

10.1128/mSphere.00370-17.1 MOVIE S1  CATH-2 instantly binds to the bacterial septum and permeabilizes the membrane. *S. aureus* live imaging was performed using 0.5 µM FITC–CATH-2 and 2.5 µM PI. DIC, FITC-labeled CATH-2 (green fluorescence), PI (red fluorescence), and merged channels are shown. One representative cell is shown. Bars = 500 nm. Download MOVIE S1, MOV file, 4 MB. Copyright © 2017 Schneider et al. 2017 Schneider et al. This content is distributed under the terms of the Creative Commons Attribution 4.0 International license.

10.1128/mSphere.00370-17.2 MOVIE S2  CATH-2 permeabilizes multiple *S. aureus* cells. Live imaging with 0.5 μM FITC-labeled CATH-2 (green) and 2.5 μM PI (red). Bars = 1 µm. Download MOVIE S2, MOV file, 2.9 MB. Copyright © 2017 Schneider et al. 2017 Schneider et al. This content is distributed under the terms of the Creative Commons Attribution 4.0 International license.

### CATH-2 causes membrane ruffling and ribosome clustering.

**FIG 3:**
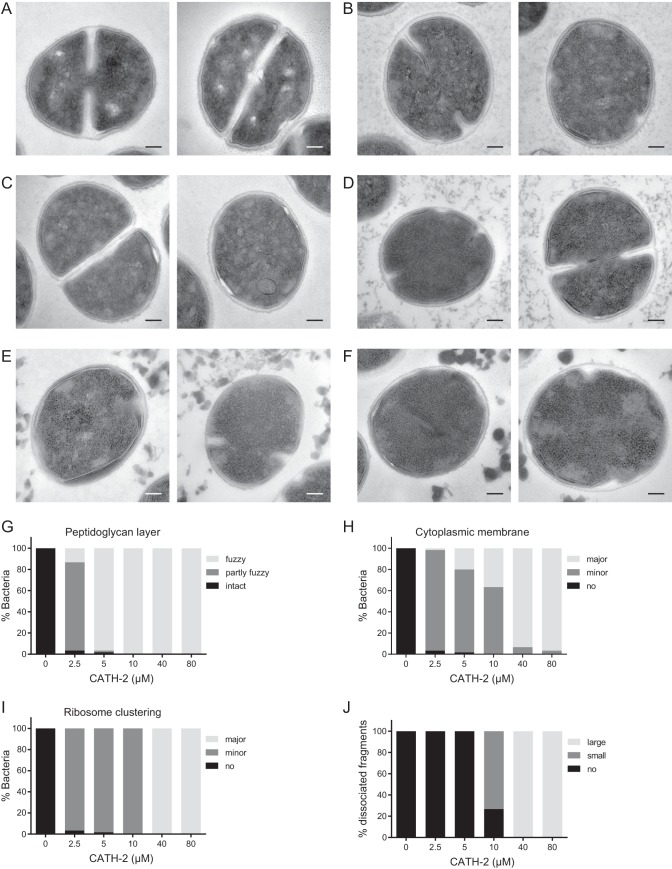
TEM imaging shows CATH-2-induced morphological changes of *S. aureus*. (A to F) Two representative images each are shown for results at concentrations of 0 µM (A), 2.5 µM (B), 5 µM (C), 10 µM (D), 40 µM (E), and 80 µM (F). (G to J) Categorization of morphological changes for peptidoglycan layer (G), membrane ruffling (H), ribosomal changes (I), and dissociated fragments (J). Bars = 100 nm.

The concentration-dependent effects of CATH-2 were determined by performing TEM studies. Representative pictures of the observed morphological changes at each CATH-2 concentration are shown in Fig. 3A to F, while quantification of the morphological changes is shown in Fig. 3G to J. At the lowest concentration used (2.5 µM), the peptidoglycan layers became fuzzy and the membrane started to ruffle. Furthermore, structural changes of the ribosomes were observed compared to the ribosome structure of the untreated control cells (Fig. 3B and G to J). At 5 µM and 10 µM, 20% and 40% of the bacteria showed major membrane ruffling, respectively, as well as minor ribosome clustering (Fig. 3C, D, H, and I). Additionally, at 10 µM, CATH-2 induced the dissociation of small bacterial fragments, which became larger and thicker at higher concentrations (Fig. 3J). At and above the MBC (≥40 µM), pronounced effects on the cell wall were observed, with major invaginations of the membrane (Fig. 3H), while intracellular ribosomal clustering also became more dense (Fig. 3I). Taken together, these results suggest that CATH-2 both attacks the membrane and alters intracellular morphology.

### CATH-2 binds the bacterial membrane and enters *S. aureus* at sub-MBCs.

**FIG 4:**
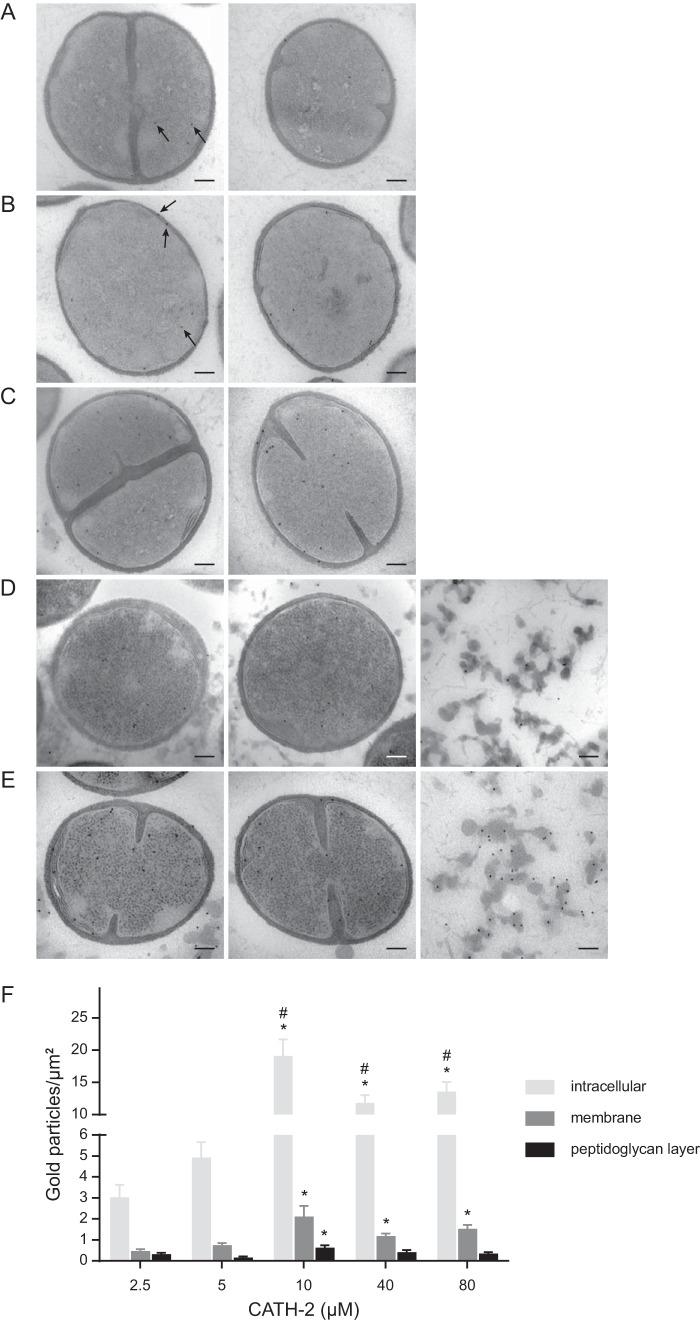
Localization of CATH-2 determined with immunogold labeling and quantification of gold particles (black arrows). (A to E) For each peptide concentration, two representative TEM images of *S. aureus* cells are shown. Peptide concentrations were 2.5 µM (A), 5 µM (B), 10 µM (C), 40 µM (D), and 80 µM (E). (D and E) The third images show gold particles on dissociated fragments when peptide concentrations at and above the MBC (≥40 µM) were used. Bars = 100 nm. (F) The number of gold particles per µm^2^ is categorized as intracellular or associated with either the membrane or the peptidoglycan layer. The data are mean values ± SEM. *, significant difference from the result for 2.5 µM (*P* < 0.001); #, significant difference from the result for 5 µM (*P* < 0.001). All peptide-treated samples were corrected for background staining.

To obtain more insights on the mechanism of action of CATH-2 against *S. aureus*, immunogold labeling of the TEM sections with a CATH-2-specific antibody was performed (Fig. 4A to F). Interestingly, at a concentration as low as 2.5 µM, considerable numbers of gold particles were detected intracellularly, and this accumulation increased significantly at higher peptide concentrations. In addition, at 2.5 μM, binding of CATH-2 to the peptidoglycan layer and the membrane was observed (Fig. 4A and F). With increasing CATH-2 concentrations, increased binding of the peptide to the membrane was observed, with a higher number of gold particles on the membranes than on the peptidoglycan layer (Fig. 4F). At 10 µM, the number of intracellular gold particles peaked (19.1 gold particles/µm^2^), with slightly lower intracellular numbers observed at higher CATH-2 concentrations (11.8 and 13.6 gold particles/µm^2^ for 40 and 80 µM, respectively) (Fig. 4C to F). At and above the MBC (≥40 µM), high numbers of gold particles were also detectable on dissociated fragments, which were present throughout the section (Fig. 4D and E, third images). These fragments are most likely cytosolic remnants released upon membrane disruption, which could explain why lower numbers of intracellular gold particles were observed at 40 µM and 80 µM than at 10 µM. Taken together, these electron microscopy studies demonstrated that already at sub-MBCs, CATH-2 binds to the bacterial membrane and localizes intracellularly, where it induces ribosomal changes and lamellar mesosome-like structures.

### CATH-2 interacts with LTA and cardiolipin.

**FIG 5:**
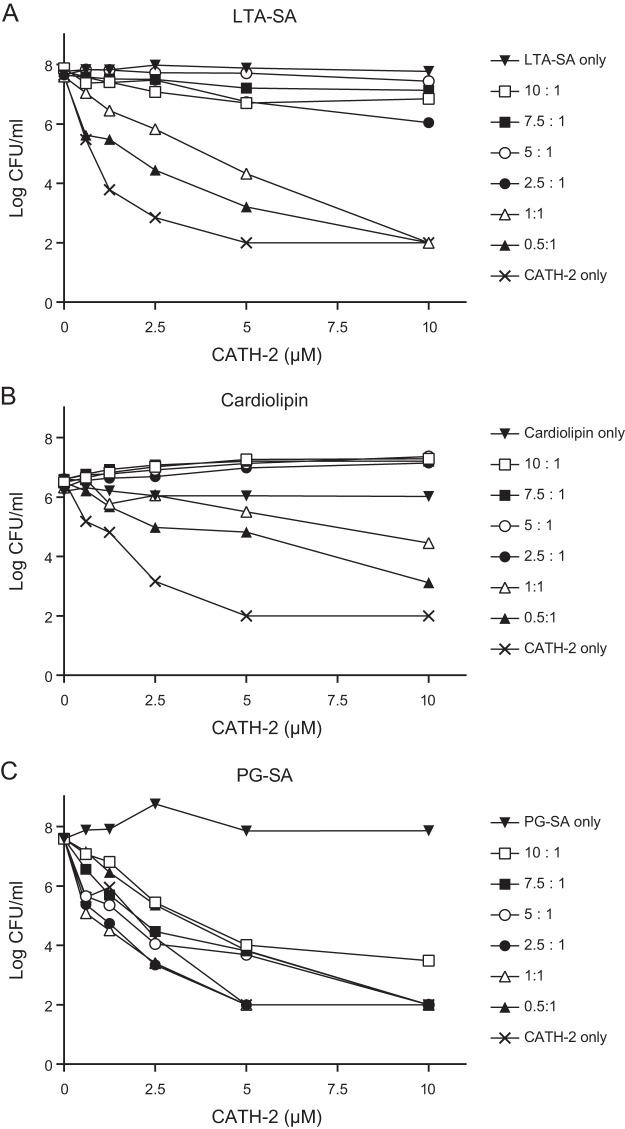
LTA and cardiolipin inhibit CATH-2-induced killing of *S. aureus*. CATH-2 was preincubated with various ratios of lipoteichoic acid from *S. aureus* (LTA-SA) (A), cardiolipin (B), and peptidoglycan from *S. aureus* (PG-SA) (C) for 1 h at 37°C. Then, mixtures were incubated with *S. aureus* for 3 h at 37°C, after which bacteria were serially diluted and spread plated on TSA plates. Viable colonies were counted after 16 h. One representative value per compound is demonstrated (*n* = 3).

**FIG 6:**
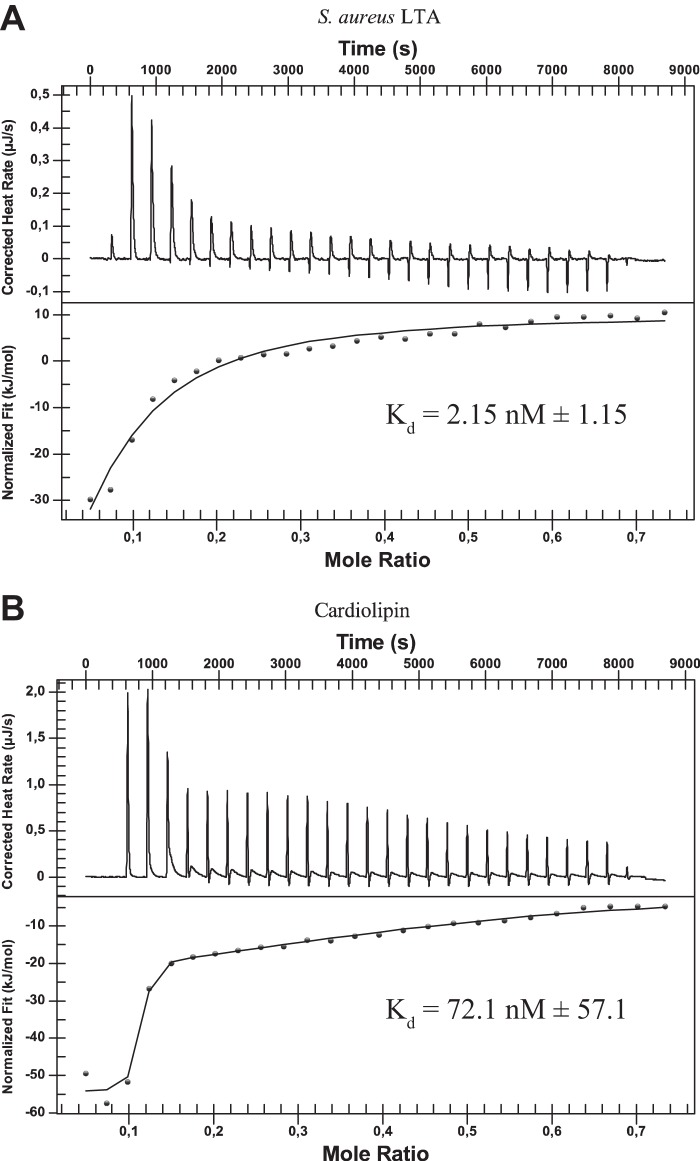
CATH-2 binds LTA and cardiolipin. Analysis of ITC titration of CATH-2 into *S. aureus* LTA (A) or cardiolipin (B) solution. Images depict representative titrations for *n* = 2 experiments. *K*_*d*_ values are the mean values ± SEM for *n* = 2 experiments.

To determine the potential interaction partners of CATH-2 on the bacterial cell wall, competition assays were performed in combination with different purified cell wall components. Peptidoglycan, LTA, and cardiolipin were preincubated in different ratios with CATH-2, after which the antibacterial activity of CATH-2 against *S. aureus* was tested. LTA and cardiolipin both strongly inhibited CATH-2-mediated *S. aureus* killing, with inhibition occurring at a ratio as low as 0.5:1 (Fig. 5A and B). In contrast, prior incubation of CATH-2 with peptidoglycan only showed a weak reduction in the killing activity of the peptide (Fig. 5C), which corresponds with our previous findings in TEM experiments, where relatively little binding of CATH-2 to the peptidoglycan layer was observed. To confirm that CATH-2 directly interacts with LTA and cardiolipin, isothermal titration calorimetry (ITC) analysis was performed. This showed that indeed CATH-2 directly bound LTA and cardiolipin. LTA was bound with an initial dissociation constant (*K*_*d*_) of 2.15 ± 1.15 nM (mean ± standard error of the mean [SEM]) (Fig. 6A; Table 1), and cardiolipin was bound with an initial *K*_*d*_ of 72.1 ± 57.1 nM (Fig. 6B; Table 1). Both interactions were enthalpy driven and showed reductions in entropy, which suggests that binding is caused by ionic interaction between the cationic CATH-2 and negative charges on LTA and cardiolipin.

**TABLE 1:** Affinity, enthalpy changes, and entropy changes for the interaction between CATH-2 and LTA or cardiolipin as determined by ITC

Parameter^a^	Mean value ± SEM for:	
	Cardiolipin	LTA
*K*_*d*_ (nM)	72.1 ± 57.1	2.15 ± 1.15
Δ*H* (kJ/mol)	−51.2 ± 3.3	−106 ± 46.4
Δ*S* (J/mol × K)	−33.7 ± 2.1	−193 ± 163

^a^ *K*_*d*_, dissociation constant, measure of affinity; Δ*H*, change in enthalpy; Δ*S*, change in entropy.

### CATH-2 inhibits *S. aureus*-induced macrophage activation at and above the MBC.

**FIG 7:**
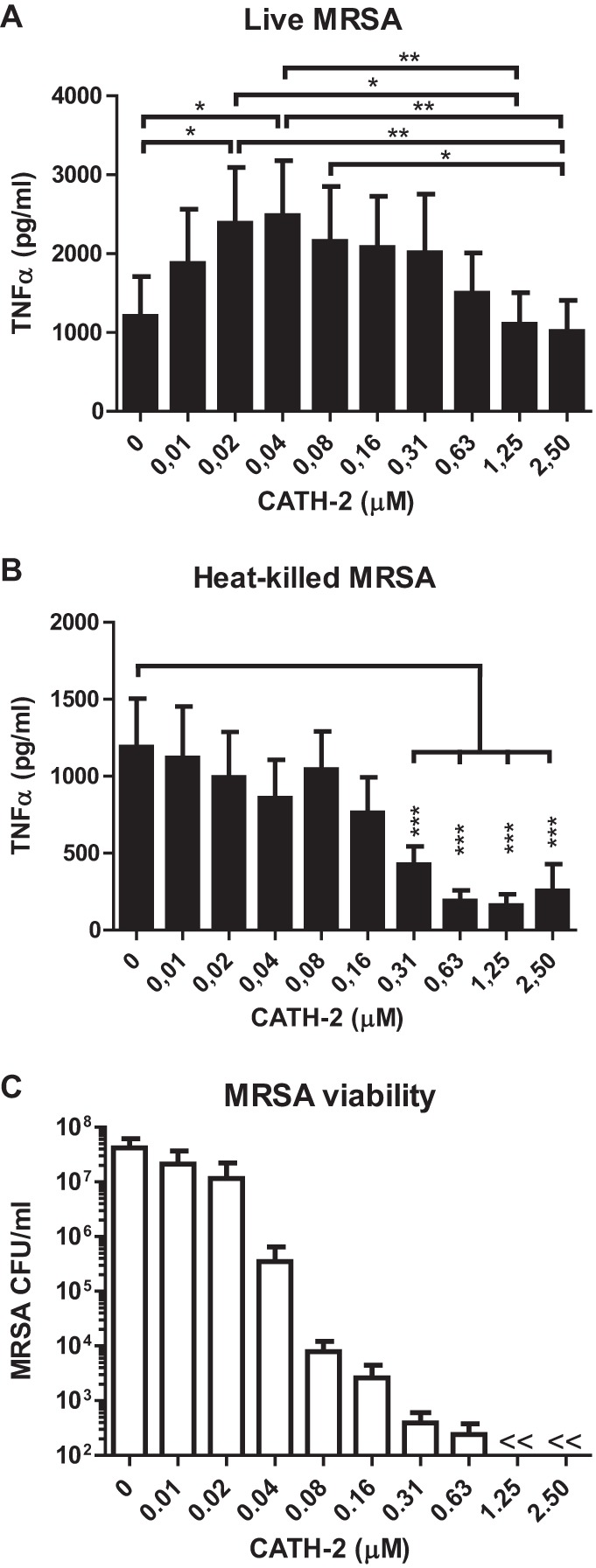
Macrophage activation by CATH-2-treated *S. aureus*. (A and B) J774.A1 macrophages were stimulated with 3 × 10^7^ CFU/ml *S. aureus*, either live (A) or heat killed (B), for 2 h in the presence or absence of indicated CATH-2 concentrations in DMEM + 10% FCS at 37°C, after which TNF-α release was determined by ELISA. (C) Colony count assay to determine *S. aureus* viability. Incubation of 3 × 10^7^ CFU/ml *S. aureus* for 2 h in DMEM + 10% FCS at 37°C in the presence or absence of indicated CATH-2 concentrations, after which bacteria were spread plated to determine viability after overnight incubation. Mean values ± SEM for *n* ≥ 3 experiments are shown. *, *P* < 0.05; **, *P* < 0.01; ***, *P* < 0.001; <<, lower than detection limit.

Recently, it was shown that CATH-2 “silently kills” *E. coli*; i.e., when CATH-2 kills *E. coli*, it also inhibits macrophage activation against the killed *E. coli* (20). To determine whether a similar effect also occurs upon killing of *S. aureus*, macrophages were stimulated for 2 h with live or heat-killed *S. aureus* in the presence or absence of various concentrations of CATH-2, after which tumor necrosis factor alpha (TNF-α) release was determined. Stimulation of macrophages with live *S. aureus* and low CATH-2 concentrations (0.02 to 0.04 μM) resulted in an increase in TNF-α release (Fig. 7A). However, when the concentration of CATH-2 was further increased, the TNF-α release was reduced back to initial (0 μM CATH-2) levels. Stimulation of macrophages with heat-killed *S. aureus* resulted in a gradual reduction of TNF-α release at higher peptide concentrations, with significant inhibition between 0.31 and 2.5 μM (Fig. 7B). In addition, as these experiments were performed in Dulbecco modified Eagle medium (DMEM) plus 10% fetal calf serum (FCS), the antimicrobial activity of CATH-2 was assessed under the same conditions, which showed a dose-dependent killing pattern with an MBC of 1.25 μM, which corresponds to the concentration at which CATH-2 is able to inhibit the macrophage activation by live *S. aureus* cells back to control levels (Fig. 7C). Together, these experiments demonstrate the potential of CATH-2 to dampen *S. aureus*-induced macrophage activation when the bacteria are killed.

## DISCUSSION

Previously, we demonstrated the antibacterial mode of action of CATH-2 on *E. coli* (19). However, in order to develop new broad-spectrum cathelicidin-based antibacterial compounds, both bacterial classes should be studied, as the mode of action against Gram-negative bacteria might not resemble the mode of action against Gram-positive bacteria. The present study demonstrates for the first time the antibacterial mode of action of chicken CATH-2 against Gram-positive bacteria. On the basis of various imaging methods, our data indicated that CATH-2 binds to and perturbs the membrane, with sub-MBCs of CATH-2 already leading to cytosolic peptide localization and morphological changes of ribosomes.

CATH-2 is an important host defense peptide of the avian immune system and was shown to exhibit antibacterial activity against different bacterial pathogens (e.g., *Salmonella*, extended-spectrum β-lactamase [ESBL]-producing *Enterobacteriaceae*, *Bacillus*, and *Campylobacter*) (12, 21–23). Furthermore, earlier studies demonstrated that CATH-2 rapidly kills *Salmonella enterica* serovar Enteritidis and *E. coli* (12). These results are in line with the current findings of CATH-2 killing *S. aureus* within 10 min. Additionally, this study demonstrated that bacteria incubated with low peptide concentrations were initially suppressed in their survival but recovered after 60 min and efficiently replicated again.

To elucidate the mechanism of action of CATH-2 against *S. aureus*, live-cell imaging was performed. Our data demonstrated the real-time binding of CATH-2 to the bacteria, in particular the bacterial septa, which was followed by cell shrinkage and membrane permeabilization. The preferred binding of HDPs to the bacterial septa has been observed previously (24–26) and could be the result of a higher local concentration of cardiolipin, which we observed was a direct interaction partner for CATH-2. To date only one Gram-positive bacterium has been used to study the real-time attack of an antimicrobial HDP. In that study, Barns and Weisshaar demonstrated that LL-37, the only human cathelicidin, causes cell shrinking and membrane permeabilization of *Bacillus subtilis* (27). Bacterial shrinkage was suggested to occur due to a disorder of turgor pressure and membrane potential, induced by membrane permeabilization. These results are in line with our current findings and similar to those of other experiments by our group showing the permeabilization and shrinkage of *Bacillus globigii* and Bacillus subtilis during CATH-2 exposure (unpublished data).

Our TEM studies showed that at a low sub-MBC (2.5 µM) of CATH-2, numerous morphological changes took place, which became more pronounced with increasing concentrations. Two main effects of CATH-2 on the bacteria were the ruffling of the membrane and the structural changes of the ribosomes. These membrane alterations are similar to the previously described effects of CATH-2 on the membrane of *Candida albicans* (28), where CATH-2 treatment triggered plasma membrane detachment from the fungal cell wall and the formation of pockets. The membrane of Gram-positive bacteria has been shown to play a distinctive role in the antibacterial mechanism of action of antimicrobial peptides. Several studies have revealed the formation of mesosomes (membrane inclusions and folding) due to peptide exposure (29–33). Studies on rat defensins and human neutrophil defensin-1 (HNP-1) showed that these peptides (600 µg/ml) target the membranes of *S. aureus* and induce lamellar mesosome formation. Interestingly, the exposure to higher defensin concentrations (3.2 mg/ml, close to MBCs) did not enhance the formation of these mesosomal structures in *S. aureus* (33). These results are in contrast with our findings, which show a CATH-2 concentration-dependent effect on the formation of mesosomes. At low peptide concentrations, punctuated invaginations were observed, whereas incubating bacteria with MBCs resulted in major lamellar mesosome formation. These differences most likely reflect different modes of action between HNP-1-mediated killing of *S. aureus* and the CATH-2-mediated killing visualized in this study.

The observation of morphological changes in *S. aureus* cells correlated with the observed intracellular localization and membrane binding of CATH-2 at sub-MBCs. Comparable findings with small (~15-amino-acid) alpha-helical peptides (MAw-1) were obtained by Azad and colleagues (34). These peptides were shown to cause deformation of the membrane, the peptidoglycan layer, and the outer acidic surfaces. Based on immunolabeling of the TEM sections, it was observed that the peptides are localized next to the induced membrane folds, suggesting that these membrane folds are aggregates of toroid-like pores produced earlier (34). This might also occur in the presence of CATH-2, as with lower peptide concentrations, similar binding of peptides near folds was detected. Our TEM studies also demonstrated that at 2.5 µM, the peptides induced a fuzzy peptidoglycan layer. However, competition assays and immuno-TEM studies demonstrated that peptidoglycan interacts only poorly with the CATH-2 peptide, suggesting that direct interaction between CATH-2 and peptidoglycan plays only a minor role in the antibacterial mode of action of CATH-2. In contrast, competition assays with LTA and cardiolipin showed that the antibacterial activity of CATH-2 was strongly inhibited by preincubation of the peptide with either compound. ITC analysis further confirmed the direct interaction of CATH-2 with LTA and cardiolipin, indicating that these negatively charged molecules most likely play a role in the initial interaction between *S. aureus* and CATH-2. Similarly, other antimicrobial peptides have also been shown to possess similar LTA and cardiolipin binding properties (25, 35, 36).

Besides its antimicrobial activity, CATH-2 has been shown to limit macrophage activation against *E. coli* and *S*. Enteritidis at MBCs (20). This means that once these Gram-negative bacteria are killed, CATH-2 can neutralize immune activation against these bacteria to prevent unnecessary inflammation. Here, we show that CATH-2 can have a similar effect on *S. aureus*, where a reduction of TNF-α release was observed at and above the MBC of CATH-2 compared to the levels of TNF-α released at sub-MBCs. Furthermore, CATH-2 was able to reduce TNF-α release when bacteria were heat killed prior to the addition of CATH-2, suggesting a potential inhibitory role for CATH-2 on immune activation by killed *S. aureus*. The observed inhibition could be the result of the neutralization of the *S. aureus*-derived LTA, as CATH-2 and other HDPs have previously been shown to prevent LTA-induced macrophage activation (9, 14, 35, 37). Interestingly, at low CATH-2 concentrations, where only limited killing was observed, CATH-2 increased TNF-α release. As *S. aureus* is a notorious evader of the immune system (38–40), it is possible that in the absence of CATH-2, the evasion strategies of *S. aureus* prevent full macrophage activation. The presence of CATH-2 might obstruct this evasion, as the bacteria require an acute defense against the peptide, which could come at the expense of immune evasion. From a host point of view, increased inflammation at sub-MBCs could also be beneficial to better detect *S. aureus*, as well as to strengthen the immune response until *S. aureus* is completely neutralized.

In conclusion, our findings provide important insights on the mechanism of action of CATH-2 against *S. aureus*. By using a unique combination of time-lapse imaging and (immuno-)TEM imaging, CATH-2 was found to cause membrane perturbation, possibly due to the interaction with negatively charged surface molecules, such as LTA and cardiolipin, which resulted in the formation of lamellar mesosomes and membrane permeabilization. However, the targeting of intracellular components at sub-MBCs that results, *inter alia*, in ribosome clustering may contribute to killing of the bacteria. Finally, we demonstrate that CATH-2-mediated killing of *S. aureus* can limit macrophage activation, which could be an important function in the regulation of inflammation during the host’s antimicrobial response against *S. aureus*. Taken together, these results provide insight into the antimicrobial mechanisms of CATH-2 against Gram-positive bacteria and contribute to a better understanding of HDP functions during infections and the development of effective HDP-based anti-infectives.

## MATERIALS AND METHODS

### Peptide.

CATH-2 (H_2_N-RFGRFLRKIRRFRPKVTITIQGSARF-NH_2_) was synthesized by 9-fluorenylmethoxy carbonyl (Fmoc) chemistry at CPC Scientific (Sunnyvale, CA). N-terminally labeled FITC–CATH-2 was synthesized by Fmoc chemistry at the Academic Centre for Dentistry Amsterdam (Amsterdam, the Netherlands).

### Colony-counting assays.

The antibacterial activity of CATH-2 was assessed by performing colony-counting assays. A methicillin-resistant *S. aureus* strain (WKZ-2, human isolate) was grown to mid-logarithmic phase in tryptone soy broth (TSB; Oxoid Limited, Hampshire, United Kingdom), washed in TSB, and diluted to 2 × 10^6^ CFU/ml. Bacteria were exposed to different CATH-2 concentrations (0 to 40 µM) for 3 h at 37°C. Subsequently, mixtures were serially diluted and spread plated on tryptone soy agar (TSA; Oxoid Limited) petri dishes as described previously (41). Surviving bacteria were counted after 16 h of incubation at 37°C.

### Killing kinetics.

The killing kinetics of CATH-2 against *S. aureus* was assessed by incubating 550 µl peptide (0.07 to 10 µM) with an equal volume of bacteria (2 × 10^6^ CFU/ml) at 37°C for several time intervals: 1, 5, 10, 20, 30, 60, 120, and 180 min. At each time point, 100 µl was immediately spread plated on TSA plates, while 20 µl was serially diluted in minimum TSB medium (1:1,000 TSB in water) and 100 µl of each dilution was spread plated on TSA plates. After 16 h at 37°C, surviving bacteria were counted.

### Live imaging.

*S. aureus* was grown to mid-logarithmic stage, washed, and resuspended in 1 ml 5 mM HEPES buffer, pH 7.4 (Sigma-Aldrich, Zwijndrecht, the Netherlands). A drop (30 µl) of 1% low-melting agarose (type I, low electroendosmosis [EEO]; Sigma-Aldrich) each was placed in the middle of silanized coverslips (Gerhard Menzel GmbH, Braunschweig, Germany), which were previously prepared with 2% (3-aminopropyl) triethoxysilane (SAA; Sigma-Aldrich) in acetone. Next, 30 μl of *S. aureus* suspension was resuspended in the agarose drop. A sandwich was made by adding a second silanized coverslip on top of the first one. After 2 min, the second coverslip was removed, and a homogenous agarose-bacterium layer was formed. Coverslips were fixed in an Attofluor cell chamber and covered in 450 µl HEPES buffer containing propidium iodide (PI; 2.5 µM final concentration). When cells were in focus, the recording was started and FITC–CATH-2 (0.5 µM final concentration) was added. Time-lapse imaging was performed using Nikon A1R and Leica SPE-II confocal microscopes at the Center for Cell Imaging (CCI) at the Faculty of Veterinary Medicine in Utrecht. Nikon NIS-Elements software was used for data analysis.

### TEM.

Transmission electron microscopy (TEM) was used to study the CATH-2-induced morphological changes of *S. aureus* in detail. For TEM experiments, 5 × 10^8^ CFU/ml *S. aureus* WKZ-2 was incubated with various concentrations of CATH-2 (0, 2.5, 5, 10, 40, and 80 µM). Due to the higher bacterial concentration required for TEM experiments, a higher MBC of 40 μM was observed. After 30 min of incubation at 37°C, the reaction was stopped by adding fixation buffer containing 2% glutaraldehyde (Polysciences, Eppelheim, Germany), 5 mM CaCl_2_, 10 mM MgCl_2_ (both from Merck, Darmstadt, Germany) in 0.1 M sodium cacodylate buffer (Sigma-Aldrich), pH 7.4. After overnight fixation at 4°C, samples were washed 3 times for 10 min each time in 0.1 M sodium cacodylate buffer, embedded in 4% low-melting-point agarose (Sigma-Aldrich), and subsequently post-fixed with 4% osmium tetroxide (Electron Microscopy Sciences, Hatfield, PA, USA), 1.5% K_4_Fe(CN)_6_ ⋅ 3H_2_O (Merck) in distilled water for 2 h at 4°C. Samples were again rinsed 3 times for 10 min and then stained with uranyl acetate for 1 h at 4°C. Subsequently, samples were extensively washed (5 times for 10 min) and embedded in Epon resin. Sections of 50 nm were mounted on 100-mesh formvar–carbon-coated copper grids and stained with uranyl acetate and lead citrate based on a Leica AC20 system (Leica, Vienna, Austria). Cells were visualized on an FEI Tecnai 12 electron microscope (FEI, Eindhoven, the Netherlands) at 80 kV.

### Immuno-TEM.

In order to determine the localization of CATH-2 on/in bacteria, the same Epon blocks previously used for the morphology determination were used. Sections (50 nm) mounted on formvar-coated copper grids were incubated with PBS containing 0.5% fish skin gelatin (Sigma-Aldrich) and 0.1% BSA-c (chemically modified bovine serum albumin; Aurion, Wageningen, the Netherlands) and immunolabeled as described previously (42). Subsequently, each section was incubated with CATH-2 antibody (43) for 1 h at room temperature (RT). After rinsing (5 times for 2 min each) with PBS, specimens were exposed to protein-A–gold particles (10 nm; Department of Cell Biology, University Medical Center Utrecht, the Netherlands) for 20 min at RT. Finally, sections were stained with 2% uranyl oxalic acetate (pH 7) (SPI, West Chester, PA, USA) for 5 min at RT and incubated with methylcellulose-uranyl acetate (pH 4, 2% methylcellulose [Sigma-Aldrich] and 4% uranyl acetate [SPI] in distilled water). Morphological changes and peptide localization were determined using a total of 60 randomly selected cells from two independent experiments. Gold particle localization was classified in three different positions—intracellular, membrane, and peptidoglycan layer—according to the methodology of Griffiths (44), and corrected for background staining using the results from control non-peptide-treated cells.

### Competition assays.

To determine potential binding targets of CATH-2 on the *S. aureus* surface, competition assays were performed. For this, concentrations of 20 to 400 µM cardiolipin (Sigma-Aldrich), lipoteichoic acid from *S. aureus* (InvivoGen, Toulouse, France), and peptidoglycan from *S. aureus* (InvivoGen) were preincubated with 40 µM CATH-2 (resulting in binding target/CATH-2 ratios of 0.5:1 to 10:1) for 1 h at 37°C. Subsequently, 2-fold dilutions of the mixtures were exposed to bacterial cultures, as described in “Colony-counting assays.” The following day, bacterial survival was assessed.

### ITC.

Isothermal titration calorimetry (ITC) was performed with the low-volume nano ITC calorimeter (TA Instruments-Waters LLC, New Castle, DE, USA). The 50-μl syringe was filled with 200 μM CATH-2 in PBS for titration into 190 μl 100 μM *S. aureus* LTA (InvivoGen) in PBS. Alternatively, the 50-μl syringe was filled with 400 μM CATH-2 in PBS, 1.5% ethyl alcohol (EtOH) for titration into 190 μl 200 μM cardiolipin (Sigma-Aldrich) in PBS, 1.5% EtOH. Titrations were incremental, with 2-μl injections at 300-s intervals. Experiments were performed at 20°C. Data were analyzed with the NanoAnalyze software (TA Instruments-Waters LLC).

### Macrophage stimulation.

For macrophage stimulation, 7.5 × 10^4^ cells/well of J774.A1 murine macrophages (45) (a kind gift of Jos van Putten, Utrecht University) were seeded in DMEM (Thermo Fisher Scientific, Waltham, MA, USA) supplemented with 10% FCS (Bodinco B.V., Alkmaar, the Netherlands) at 37°C, 5.0% CO_2_. After overnight adherence, cells were stimulated with live or heat-killed (1 h at 80°C) *S. aureus* (multiplicity of infection [MOI] of 20:1) in the presence or absence of CATH-2 (0.01 to 2.5 µM) for 2 h at 37°C in DMEM plus 10% FCS, after which supernatants were collected to determine TNF-α levels by enzyme-linked immunosorbent assay (ELISA; R&D Systems Minneapolis, MN). TNF-α ELISAs were performed according to the manufacturer’s protocol. Samples were measured with a FLUOstar omega microplate reader (BMG Labtech GmbH, Ortenberg, Germany) and analyzed with MARS data analysis software (BMG Labtech GmbH). Measurements of optical density at 450 nm (OD_450_) were corrected by subtracting OD_570_ measurements. In addition to macrophage stimulation, mixtures of *S. aureus* and CATH-2 in DMEM plus 10% FCS were incubated for 2 h at 37°C, followed by serial dilution and spread plating on tryptone soy agar petri dishes to determine bacterial viability after overnight incubation.

### Statistical analysis.

For statistical analysis, SPSS 22 software (IBM, Armonk, NY, USA) was used. Analysis of immunogold-labeling distribution was performed with the nonparametric Kruskal-Wallis test. Analysis of differences in TNF-α release was determined by repeated one-way analysis of variance (ANOVA) with Tukey’s *post hoc* correction.
